# Persistent median artery inside the carpal tunnel: description and surgical implications

**DOI:** 10.4322/acr.2020.209

**Published:** 2020-09-02

**Authors:** João Gabriel Alexander, Matheus Coelho Leal, Josemberg da Silva Baptista

**Affiliations:** 1 Universidade Federal do Espírito Santo (UFES), Departamento de Morfologia, Laboratório de Estudos em Morfologia Aplicada (LEMA), Vitória, ES, Brasil.

**Keywords:** Anatomy, regional, Biological Variation, Individual, Carpal Tunnel Syndrome, Nerve Compression Syndromes

## Abstract

The median artery is usually a transient vessel during the embryonic period. However, this artery can persist in adult life as the persistent median artery. This paper aims to describe this relevant anatomical variation for surgeons, review the literature and discuss its clinical implications. A routine dissection was performed in the upper left limb of a male adult cadaver of approximately 50-60 years of age, embalmed in formalin 10%. The persistent median artery was identified emerging as a terminal branch of the common interosseous artery with a path along the ulnar side of the median nerve. In the wrist, the persistent median artery passed through the carpal tunnel, deep in the transverse carpal ligament. The dissection in the palmar region revealed no anastomosis with the ulnar artery forming the superficial palmar arch. The common digital arteries emerged from the ulnar artery and the persistent median artery. Such variation has clinical and surgical relevance in approaching carpal tunnel syndrome and other clinical disorders in the wrist.

## INTRODUCTION

The median artery is usually a transient vessel of the early embryonic period, responsible for blood irrigation of the embryo's hand. Its regression occurs around the eighth week of gestation, when the radial and ulnar arteries are developed.^1^ ^-^ ^3^ However, in some cases, this artery can persist in adulthood life in two distinct patterns: (1) the antebrachial type, which represents a partial involution of the embryonic artery, and in which the artery ends before reaching the wrist; (2) the palmar type, in which the artery persists similarly to the embryonic pattern, being larger and longer, and reaching the palm. The term persistent median artery (PMA) refers to the second pattern.^3^ ^,^ ^4^

Sir Richard Quain (1816-1898) was the first to describe the PMA, in 1844, as a small artery deriving from the brachial artery in the cubital fossa and joining the course of the median nerve (MN).^5^ Subsequently, cases of PMA with a prevalence of a varied spectrum ranging from 0.6% to 30% were described in the literature, especially in cadavers.^6^ This anatomical variation remains asymptomatic, in most cases.^7^ However, it may be associated with carpal tunnel syndrome (CTS), especially when related to PMA thrombosis, aneurysm or median nerve compression.^8^ ^,^ ^9^

Several studies have described such anatomical variation. Nevertheless, PMA varies both in its origin and in its topography along the forearm, carpal tunnel and hand, making such variation pertinent for physicians during their clinical and surgical practice.

The present work aimed to describe this relevant anatomical variation, review the literature and discuss its clinical implications.

## CASE REPORT

The left upper limb dissection of a male cadaver of approximately 50-60 years of age, fixed in 10% formalin. A layer-by-layer routine dissection was performed removing the skin and the subcutaneous tissue of the cubital fossa, forearm, carpal tunnel and palmar region. First, by dissecting the cubital fossa, the PMA was identified emerging as a terminal branch of the common interosseous artery. Second, the forearm muscles and the arteries (the anterior, posterior and recurrent interosseous artery, and the radial and ulnar artery) were all isolated, and emphasis was given to the median nerve (MN) and the PMA, following their path. Third, dissection in the palmar region was performed focusing on vascular structures and their topography to observe the superficial palmar arch (SPA) pattern and its emergent branches. Finally, a dissection on the transverse carpal ligament (TCL) was conducted by a meticulous sagittal section. The results are described with photographic record of the dissections.

## AUTOPSY PRESENTATION

The PMA was identified in the cubital fossa emerging as one of the terminal branches of the common interosseous artery, passing through the pronator teres muscle and over the MN, reaching the ulnar or medial side of the MN after the cubital fossa. In the rest of the forearm, the PMA remained associated along the ulnar/medial side of the MN, deep in the palmaris longus muscle, in between the flexor digitorum superficialis muscles and the flexor pollicis longus muscle, and superficial to the flexor digitorum profundus muscle.

In the wrist, both the PMA and MN superficialized, in a way that the PMA admitted an anteromedial position concerning MN. Through the carpal tunnel, the PMA and MN ran underneath the TCL, medially to the tendon of the flexor pollicis longus muscle and laterally to the flexor digitorum superficialis muscle.

**Figure 1:**
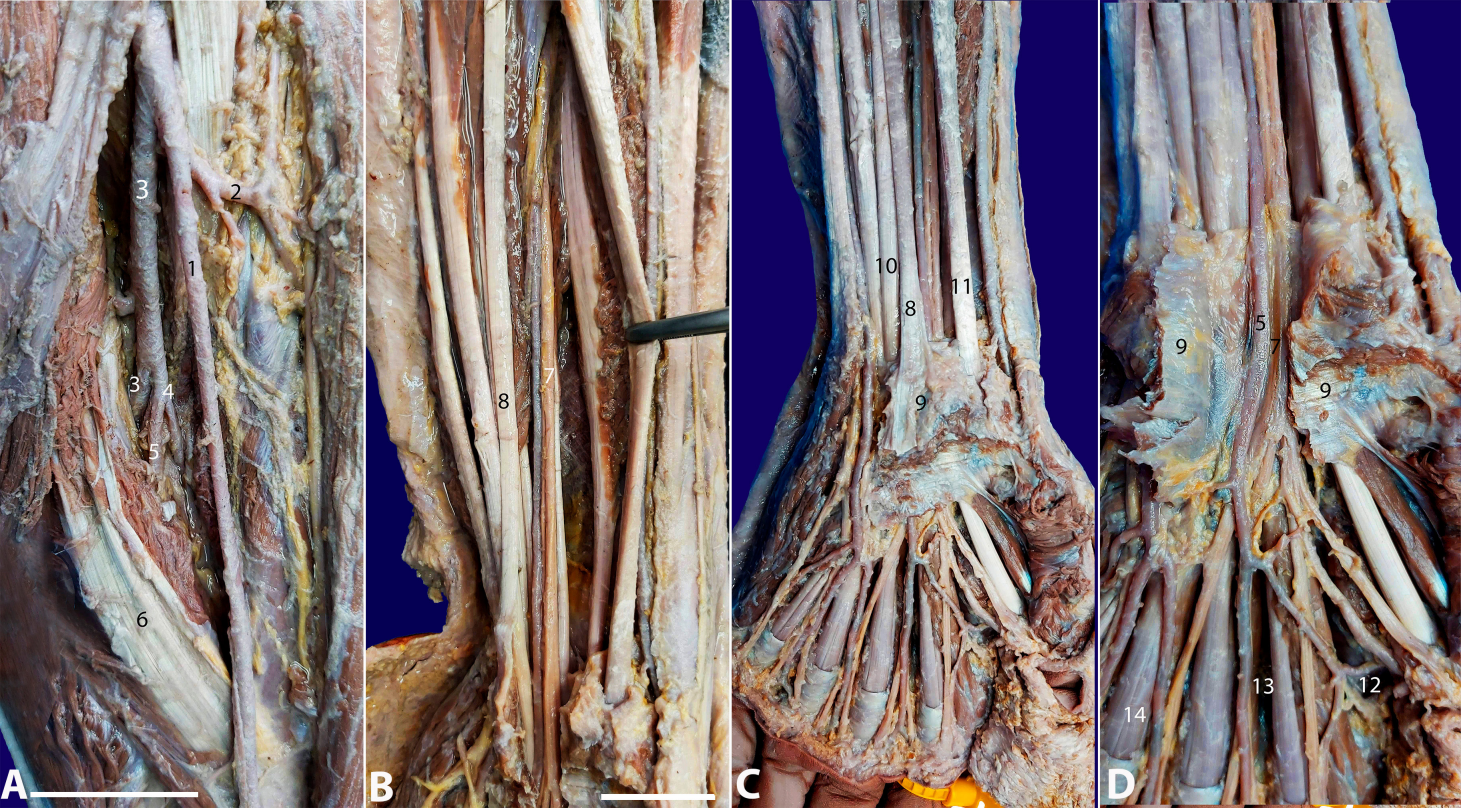
*Photographs of the case.* **A –** Cubital fossa; **B –** Forearm presentation; **C –** Wrist presentation; **D –** Opened transverse carpal ligament: 1) radial artery; 2) radial recurrent artery; 3) ulnar artery; 4) common interosseous artery; 5) persistent median artery; 6) pronator teres muscle; 7) median nerve; 8) palmaris longus tendon; 9) transverse carpal ligament (flexor retinaculum); 10) flexor digitorum superficialis tendons; 11) flexor pollicis longus tendon; 12) radial artery in anastomosis with the common digital artery of the index finger; 13) common digital palmar artery from the persistent median artery; 14) common digital palmar arteries from the ulnar artery. Scale bar A) and D) are 3cm; B) and C) are 5cm.

In the palmar region, the PMA crossed over the MN to originate the common digital artery of the index and middle fingers. No anastomosis forming the SPA was observed. The radial artery was visualized ending in anastomosis with the common digital artery of the index finger. The common digital arteries emerged from the ulnar artery and the PMA in association with the common digital nerves. No further variations were observed (Figure 1).

## **D**ISCUSSION

Variations in the origin, path and termination of PMA have been described over the past decades by a large number of reports emphasizing the association of this variation with clinical procedures, particularly CTS. Several factors may be related to the PMA: cell adhesion molecules, transcription factors, mechanical forces, and vascular regression and remodeling. All such factors are involved in the early embryogenesis stages or the formation of the initial vascular architecture. Thus, the persistence of this architecture explains the PMA in adulthood.^10^

The PMA predominance in the limb, whether unilateral or bilateral, is controversial. While Pierre-Jerome et al. described that the PMA was more frequent bilaterally, and when unilateral, it was more frequent in the left limb of females,^11^ Chen et al. reported that there is no statistical difference regarding the side of occurrence.^12^ Furthermore, bilateralism was not verified in the present study.

The PMA can originate from the ulnar, radial, common or anterior interosseous, or brachial arteries.^11^ Two patterns are described in the literature: 1) PMA would originate from the common caudal angle, between the ulnar artery and the common interosseous trunk, and anastomosis with the ulnar artery in the hand, would form the SPA; 2) PMA would most often originate from the anterior interosseous artery, not forming the SPA.^11^ The present case is very similar to the second pattern, considering the absence of the SPA and anastomosis with the ulnar artery. However, it originated from the common interosseous artery.

The topography of the PMA and MN along the forearm in the present case corroborates Chen et al. and seems to be very constant.^12^ Nevertheless, such topography changes in the carpal tunnel in a variable way: anterior, anterolateral or anteromedial position, which is similar to the present case.^10^ ^,^ ^12^ Thus, this makes a preoperative ultrasound an essential tool to avoid iatrogenic injuries during the surgical procedure in the CTS.^12^

The description of the PMA terminal branches takes into consideration the formation of the SPA and the emergence of the common digital arteries. SPA is made by anastomosis between the PMA and the ulnar artery, being considered a complete pattern or so-called “medial-ulnar type”. The incomplete pattern has no SPA formation, no anastomosis with ulnar artery, and only common digital arteries emerge as terminal branches of the PMA, exactly as the present case.^10^ ^,^ ^13^ ^,^ ^14^ Although such patterns are well described, the frequency is not precise. Hand surgeons would be aware of this unusual topography.^10^

CTS is the most common entrapment neuropathy. Caused by compression of the MN in the wrist, it is related to several etiologies that increase the content and compromise the capacity of the carpal tunnel.^15^ Salter et al. described the PMA thrombosis as one of the etiologies of CTS due to the pressure exerted in the carpal tunnel.^7^ This thrombosis could be caused by different factors, such as infection on deep fascial planes, work in an unusual position of the wrist, trauma, hormonal contraceptive pills, and exhaustive work on wrist.^7^ Other possible etiologies related to PMA would also be associated with CTS, including calcification, atherosclerosis, aneurysm, and trauma.^13^ Moreover, PMA could be related to other clinical disorders, such as anterior interosseous nerve syndrome and pronator syndrome.^16^

## CONCLUSION

This work has described an important case of the PMA, drawn attention to its clinical and surgical relevance, and reviewed the literature. Such variation has important clinical implications in CTS, as well as in anterior interosseous nerve syndrome and pronator syndrome. The preoperative plan containing images of exams is essential to avoid iatrogenic lesions which can compromise the median nerve and the hand blood flow. Thus, knowing this anatomy and the possible variations of PMA proves relevant for clinical and surgical practice.
